# Severe COVID-19 and long COVID are associated with high expression of *STING*, *cGAS* and IFN-α

**DOI:** 10.1038/s41598-024-55696-0

**Published:** 2024-02-29

**Authors:** Maria Alice Freitas Queiroz, Wandrey Roberto dos Santos Brito, Keise Adrielle Santos Pereira, Leonn Mendes Soares Pereira, Ednelza da Silva Graça Amoras, Sandra Souza Lima, Erika Ferreira dos Santos, Flávia Póvoa da Costa, Kevin Matheus Lima de Sarges, Marcos Henrique Damasceno Cantanhede, Mioni Thieli Figueiredo Magalhães de Brito, Andréa Luciana Soares da Silva, Mauro de Meira Leite, Maria de Nazaré do Socorro de Almeida Viana, Fabíola Brasil Barbosa Rodrigues, Rosilene da Silva, Giselle Maria Rachid Viana, Tânia do Socorro Souza Chaves, Adriana de Oliveira Lameira Veríssimo, Mayara da Silva Carvalho, Daniele Freitas Henriques, Carla Pinheiro da Silva, Juliana Abreu Lima Nunes, Iran Barros Costa, Izaura Maria Vieira Cayres-Vallinoto, Igor Brasil-Costa, Juarez Antônio Simões Quaresma, Luiz Fábio Magno Falcão, Eduardo José Melo dos Santos, Antonio Carlos Rosário Vallinoto

**Affiliations:** 1 Laboratory of Virology, Institute of Biological Sciences, Federal University of Pará, Belém, Brazil; 2 Graduate Program in Biology of Infectious and Parasitic Agents, Institute of Biological Sciences, Federal University of Pará, Belém, Brazil; 3 Laboratory of Genetics of Complex Diseases, Institute of Biological Sciences, Federal University of Pará, Belém, Brazil; 4 Laboratory of Basic Research On Malaria, Parasitology Section, Evandro Chagas Institute, Health and Environment Surveillance Secretariat, Brazilian Ministry of Health, Ananindeua, Brazil; 5 School of Medicine, Institute of Medical Sciences, Federal University of Pará, Belém, Pará Brazil; 6 Belém Adventist Hospital, Belém, Brazil; 7 Arbovirology and Hemorrhagic Fevers Section, Evandro Chagas Institute, Health and Environment Surveillance Secretariat, Brazilian Ministry of Health, Ananindeua, Brazil; 8 Laboratory of Immunology, Section of Virology, Instituto Evandro Chagas, Health and Environment Surveillance Secretariat, Brazilian Ministry of Health, Ananindeua, Brazil; 9 Graduate Program in Virology, Evandro Chagas Institute, Department of Science, Technology, Innovation and Strategic Health Inputs, Ministry of Health of Brazil, Ananindeua, Brazil; 10 Center of Biological and Health Sciences, University of the State of Pará, Belém, Brazil

**Keywords:** Immunology, Virology

## Abstract

The cGAS-STING pathway appears to contribute to dysregulated inflammation during coronavirus disease 2019 (COVID-19); however, inflammatory factors related to long COVID are still being investigated. In the present study, we evaluated the association of *cGAS* and *STING* gene expression levels and plasma IFN-α, TNF-α and IL-6 levels with COVID-19 severity in acute infection and long COVID, based on analysis of blood samples from 148 individuals, 87 with acute COVID-19 and 61 in the post-COVID-19 period. Quantification of gene expression was performed by real-time PCR, and cytokine levels were quantified by ELISA and flow cytometry. In acute COVID-19, *cGAS*, *STING*, IFN-α, TNF-α, and IL-6 levels were higher in patients with severe disease than in those with nonsevere manifestations (*p* < 0.05). Long COVID was associated with elevated *cGAS*, *STING* and IFN-α levels (*p* < 0.05). Activation of the cGAS-STING pathway may contribute to an intense systemic inflammatory state in severe COVID-19 and, after infection resolution, induce an autoinflammatory disease in some tissues, resulting in long COVID.

## Introduction

Coronavirus disease 2019 (COVID-19) involves different clinical manifestations, ranging from mild and moderate forms to the most severe form of the disease, which is characterized by severe acute respiratory syndrome (SARS), with a high risk of death^1,2^.

Several factors may favor the development of severe COVID-19, including advanced age, sex, the presence of comorbidities and altered activation of immune mechanisms, which may contribute to increased replication of severe acute respiratory syndrome coronavirus 2 (SARS-CoV-2) and trigger systemic immunopathological reactions^2–4^.

Another condition resulting from SARS-CoV-2 infection is long COVID, which is defined by the presence of a variety of symptoms that persist after resolution of the acute infection and are not explained by other causes; the symptoms include cognitive and mental impairments, pain in the chest and joints, palpitations, myalgia, smell and taste dysfunctions, cough, headache, and gastrointestinal and heart problems^5–7^. Individuals with long COVID may develop myalgic encephalo-myelitis/chronic fatigue syndrome (ME/CFS)-related postexertional malaise, responsible for causing a severe inability to perform common activities that healthy people realize with ease^8^. Long COVID appears to be associated with a change in the inflammatory response, which persists in some individuals, regardless of the severity of the acute COVID-19 episode^9,10^.

The innate immune system is the body's first line of defense against invading pathogens, and it is mobilized by a variety of cell receptors that detect molecular patterns composing the viral structure. However, several strategies are exploited by SARS-CoV-2 to disrupt antiviral innate immune responses^11^, including the production of viral proteins that inhibit the translocation of stimulator of interferon genes (STING), attenuating the innate antiviral response^12^.

Canonically, the cyclic GMP-AMP synthase (cGAS)-STING pathway is activated when cGAS binds to double-stranded DNA (dsDNA) or DNA:RNA hybrids in the cell cytoplasm. This event leads to the production of cyclic GMP-AMP (cGAMP), which interacts with STING and promotes its dimerization, followed by interaction with TANK-binding kinase 1 (TBK1) and the transcription factor interferon regulatory factor 3 (IRF3). STING promotes the transphosphorylation of IRF3, leading to pIRF3 formation, which then translocates to the nucleus and helps to induce IFN-I transcription^13–15^.

Although the major IFN expression-inducing pattern recognition receptors (PRRs) that detect viral RNAs are members of the retinoic acid-inducible gene I (RIG-I) and Toll-like receptor (TLR) protein families, STING also plays an important role in RNA virus infection^16^ and has been associated with infections by vesicular stomatitis virus, dengue virus, coronavirus, and influenza virus. STING may play a relevant role in these infectious processes because, similar to SARS-CoV-2, these viruses have developed strategies to antagonize cGAS-STING signaling^12,17–20^. In addition, the cGAS-STING pathway can respond to viral signatures or mediate the inflammatory process via detection of cellular DNA released from mitochondria or the nucleus resulting from stress caused by the infection^21^.

To determine the role of STING in RNA virus infection, Franz et al.^16^ evaluated a panel of viruses from different families in the presence and absence of STING and observed that all viral infections were more productive in its absence, indicating that STING is required for controlling the replication of several RNA viruses. In SARS-CoV-2 infection, activation of the cGAS-STING pathway appears to be intensified as a result of a host collateral response to tissue damage^22^.

The intensity of the immune response is a critical factor during COVID-19, and evaluation of the components that activate innate immunity mechanisms may improve understanding of how the antiviral and inflammatory responses of the host influence the development of the severe form of acute or long COVID^9,23^. Thus, the present study aimed to investigate the association between the severe form of acute COVID-19 and long COVID with the gene expression of *STING* and *cGAS* and the plasma levels of IFN-α, TNF-α and IL-6.

## Materials and methods

### Participant characteristics and sample collection

In the present study, blood samples from 148 individuals with COVID-19, were analyzed. Of all the patients, 87 had COVID-19 at the time of sample collection, that is, acute COVID-19, and were classified as having severe (n = 44) or nonsevere (n = 43) clinical manifestations according to the criteria established by the World Health Organization^1^. This study also included 61 individuals who had no active infection at the time of sample collection (the post-COVID-19 period); of these subjects, 30 had long and 31 had already recovered from COVID-19 and did not have any symptoms related to long COVID syndrome (these individuals were followed up for 6 months after infection resolution)^24^. All individuals in the post-COVID period experience mild clinical manifestations during COVID-19.

The participants included individuals of both sexes, aged 18 years or over, who were untreated, had not yet been vaccinated against SARS-CoV-2 and were treated at the COVID-19 outpatient clinic of the State University of Pará, in the Belém Adventist Hospital and at the Evandro Chagas Institute from July 2020 to May 2021. The diagnostic criteria for long-COVID consisted of: (I) Having had an acute COVID-19 diagnosis with symptoms (severe, moderate or severe) with confirmation of SARS-CoV-2 infection by real-time polymerase chain reaction amplification; (II) Have presented prolonged clinical manifestation of at least one symptom of COVID-19, such as fatigue, dyspnea, cough, chest pain, muscle pain or weakness, headache, insomnia, visual disturbances, tremor, loss of balance, edema lower limb pain, arthralgia, palate and/or smell disorders; (III) Symptoms persist for at least 3 months after resolution of the acute infection; (IV) Post-COVID-19 symptoms cannot be attributed to any other possible cause. The prevalence of symptoms related to long COVID in the investigated group is described in Table 1. These patients were treated at the outpatient clinic for long COVID at the State University of Pará^25^.

**Table 1:** Prevalence of symptoms in the long COVID group.

Long COVID	Frequency (n = 30)	
	N	%
**Symptoms**		
Dyspnea	24	80.00
Muscle weakness	22	73.33
Chest pain	12	40.00
Tremor	11	36.66
Fatigue	23	76.66
Myalgia	20	66.66
Headache	16	53.33
Insomnia	15	50.00
Visual changes	11	36.66
Lower limb edema	10	33.33
**Number of symptoms**		
2–4	6	20.00
5–7	18	60.00
8–10	6	20.00

N = Individuals number.

Individuals with an established diagnosis of other diseases (including genetic, infectious, autoimmune diseases, cancer and physical trauma) were excluded from the study.

Blood samples (10 mL) were collected by intravenous puncture using a vacuum collection system containing ethylenediaminetetraacetic acid (EDTA) as an anticoagulant. The samples were transported to the Virology Laboratory of the Federal University of Pará, where they were processed for separation of plasma and leukocytes. Leukocyte samples were used for RNA extraction, and plasma samples were used for plasma cytokine assessment^24^.

### RNA extraction and reverse transcription

Total RNA was extracted from peripheral blood leukocytes using a TRIzol™ Plus RNA Purification Kit (Thermo Fisher Scientific, Waltham, Massachusetts, USA), and all steps followed the protocol recommended by the manufacturer. The concentration of the extracted RNA was determined using a BioDrop™ (Bio-Rad, Hercules, California, USA) according to the manufacturer's instructions. The concentrations of all total RNA samples for synthesis of complementary DNA (cDNA) were equal to 50 ng/µL^25^.

The extracted RNA was converted to cDNA using a High Capacity cDNA Reverse Transcription® with RNAse Inhibitor Kit (Applied Biosystems, Foster City, CA, USA). For the cDNA reaction, a mixture was prepared with a final volume of 20.0 µL containing 2 µL of 10X RT Buffer, 0.8 µL of 25X dNTP Mix (100 nM), 2 µL of random primer, 1 µL of MultiScribe™ Reverse Transcriptase, 1 µL of RNaseOUT™ and 3.2 µL of ultra-pure water provided by the kit as well as 10.0 µL of extracted RNA. The mixture was subjected to cycles of 25 °C for 10 min, 37 °C for 120 min and 85 °C for 5 min using a Mastercycler Personal thermocycler (Eppendorf, Hamburg, Germany)^25^.

### Quantification of gene expression

Gene expression was determined by mRNA quantification using real-time PCR. Initially, standardization of qPCRs with cDNAs and probes (endogenous and target genes) was carried out to calculate the efficiency of the amplification reactions in which different concentrations of cDNA were tested (neat and in 4 dilutions of factor 2 - − 1:2, 1:4, 1:8 and 1:16). All reactions were performed in microtiter plates and in triplicate while simultaneously analyzing the same cDNA (at different dilutions) with different probes to construct an efficiency curve to validate the 2-ΔΔCT analysis method. All assays showed good efficiency, as expected (100% ± 10)^26^.

Relative quantification of gene expression consisted of amplification of the target gene with the endogenous gene (normalizer) using TaqMan™ assays (Applied Biosystems, Foster City, CA, USA) and the StepOnePLUS™ Real-Time PCR System (Thermo Fisher Scientific, Waltham, MA, USA). The reactions were performed in the singleplex format according to the manufacturer's protocol. Hs00736955_g1 was used for *STING* and Hs00403553_m1 for *cGAS*; glyceraldehyde-3-phosphate dehydrogenase (*GAPDH*) was used as an endogenous control (Hs02786624_g1). All assay kits were obtained commercially (Thermo Fisher Scientific, Waltham, MA, USA). For the reaction, 15 µL of 2X TaqMan® Universal PCR Master Mix, 1.5 µL of 20 × TaqMan Gene Expression Assay, 3 µL of cDNA and 10.5 µL of RNase-free water were used, with the following thermocycling conditions: 2 min at 50 °C, followed by 10 min at 95 °C and 1 min at 60 °C^25^.

Relative quantification (RQ) of target gene expression was determined using the comparative CT method (∆∆Ct) with the 2-ΔΔCT formula, where ∆∆Ct = ∆Ct sample–∆Ct reference (Life Technologies, Carlsbad, CA, USA)^25^.

### Plasma concentration of cytokines

Quantification of TNF-α and IL-6 levels was performed using flow cytometry with a Cytometric Bead Array (CBA) Human Th1/Th2/Th17 Kit (BD Biosciences, San Diego, CA, USA) and BD FACS Canto II equipment. All procedures followed the manufacturer's guidelines. The methodology used is based on beads conjugated with a capture antibody, in which specific populations of beads with known fluorescence intensities and conjugated to a specific capture antibody are mixed to form the CBA, and fluorescence is then measured in the FL-3 channel of the flow cytometer^24^. IFN-α levels in plasma samples were quantified using an ELISA-type immunoenzymatic assay, the Elabbscience Human IFN alpha ELISA Kit (Houston, TX, USA), according to the manufacturer's recommendations^25^.

### Statistical analysis

The information obtained was entered into a database in Microsoft Office Excel 2013 software. Normality analysis of the distribution of cytokine levels was performed using the Shapiro‒Wilk test. Based on the results of the normality test, evaluation of the plasma levels of these markers between the groups with acute, severe and nonsevere COVID-19 was performed using the nonparametric Mann‒Whitney test. A correlogram was generated to evaluate the correlation between the expression levels of cGAS and STING and the plasma levels of cytokines using the Spearman correlation test. The ROC curve was used to assess whether the levels of the investigated markers are capable of differentiating groups in acute COVID-19 (severe and non-severe) and in the post-COVID period (long COVID and non-long COVID). Tests were performed using GraphPad Prism 5.0 and RStudio 4.0.1 programs, with *p* < 0.05 considered to indicate significance.

Heatmap graphics were inferred using the R studio 4.2.1 program with the gplots, RColorBrewer and preprocessCore plotting packages; we utilized the ward d2 method as the distance type and the canberra method as the aggregation type^27^.

### Ethics declaration

The study was approved by the Brazilian National Research Ethics Committee (CAEE: 33470020.1001.0018), protocolo nº 2.190.330, according to the guidelines of CNS Resolution nº 466/2012. Research involving human participants was carried out in accordance with the Declaration of Helsinki. All individuals were informed about the research objectives, and those who agreed to participate in the study signed a free and informed consent form and responded to a clinical-epidemiological questionnaire.

## Results

### Characterization of the investigated groups

The patients with acute COVID-19 had a mean age of 52 years, and the majority were male (n = 48; 55.17%). The individuals evaluated in the post-COVID-19 period had a mean age of 40.5 years, and 35 were female (57.37%). The characteristics of each analyzed group are described in Table 2.

**Table 2:** Demographic and clinical characteristics of the participants in the acute COVID-19 and post-COVID-19 period.

Group	n (%)	n (%)
Acute COVID-19	Severe (n = 44)	Nonsevere (n = 43)
**Sex**		
Male	25 (56.82)	23 (53.49)
Female	19 (43.18)	20 (46.51)
**Age**		
21–40	6 (13.63)	13 (30.23)
41–60	21 (47.73)	23 (53.49)
> 60	17 (38.64)	7 (16.28)
**Comorbidities**		
Present	22 (50)	14 (32.56)
Absent	22 (50)	29 (67.44)

Post-COVID	Long COVID (n = 30)	No long COVID (n = 31)
**Sex**		
Male	10 (33.33)	16 (51.61)
Female	20 (66.67)	15 (48.39)
**Age**		
18–39	9 (30.00)	18 (58.06)
40–60	20 (66.67)	13 (41.94)
> 60	1 (3.33)	0 (0.00)
**Comorbidities**		
Present	11 (36.67)	5 (16.13)
Absent	19 (63.33)	26 (83.87)
**Symptoms**		
Dyspnea, muscle weakness, chest pain, tremor, fatigue, myalgia, headache, insomnia, visual changes, lower limb edema	30 (100)	0 (0.00)

n: number of individuals; comorbidities: diabetes mellitus, chronic cardiovascular disease, obesity, chronic kidney disease, lower limb edema.

### Gene expression of * cGAS* and * STING*

**Figure 1:**
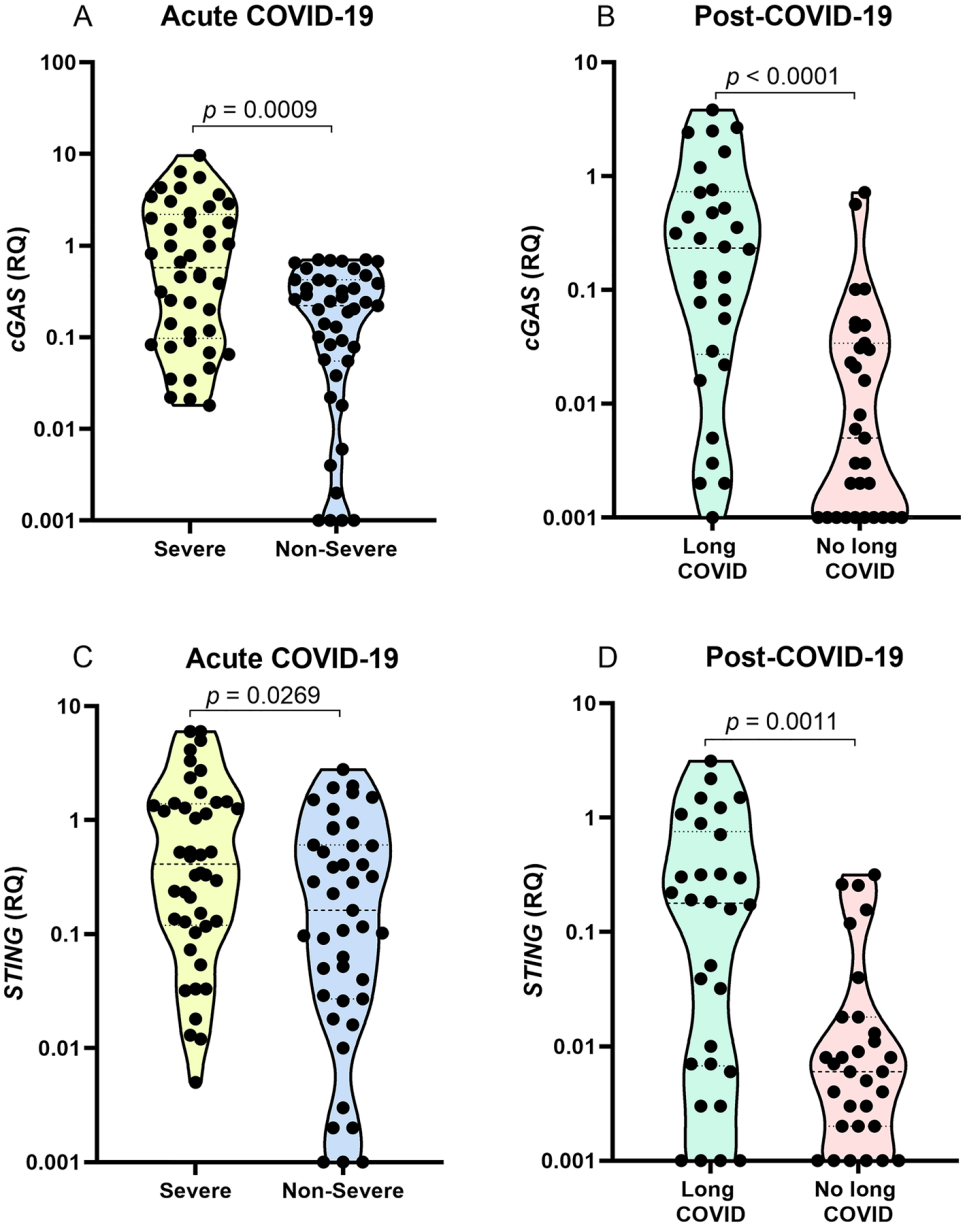
Comparison of *cGAS* gene expression levels in the (**A**) acute COVID-19 and (**B**) post-COVID-19 period groups; *STING* gene expression in the (**C**) acute COVID-19 and (**D**) post-COVID-19 period groups.

Evaluation of *cGAS* and *STING* gene expression levels showed that the group with the severe form of acute COVID-19 had higher levels of *cGAS* (*p* = 0.0009; Fig. 1A) and *STING* (*p* = 0.0269; Fig. 1C). Regarding the groups evaluated in the post-COVID-19 period, the group with long COVID had higher levels of *cGAS* (*p* < 0.0001; Fig. 1B) and *STING* (*p* = 0.0011; Fig. 1D).

### Plasma levels of cytokines

**Figure 2:**
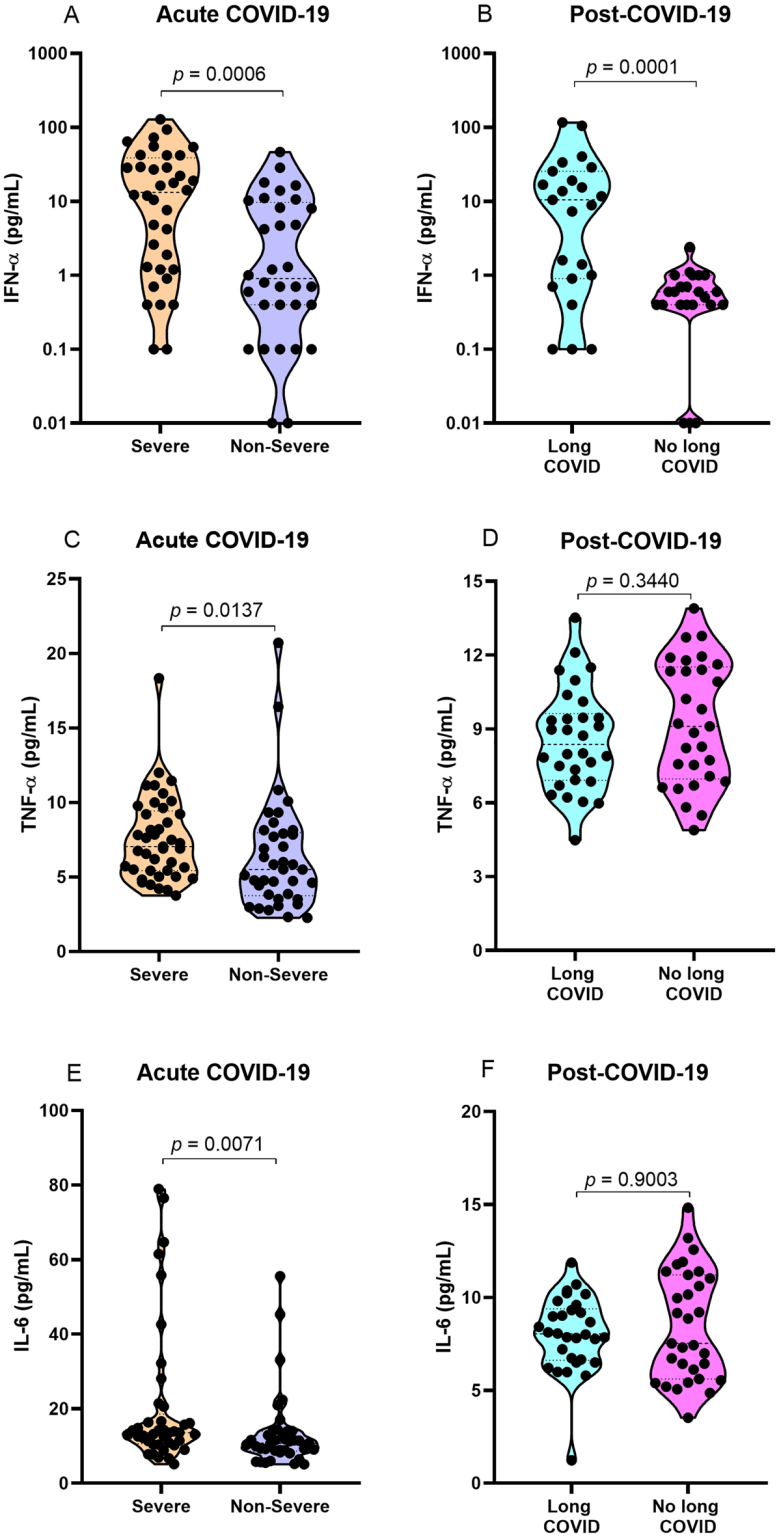
Comparison of the plasma levels of the cytokines (**A**) IFN-α, (**C**) TNF-α and (**E**) IL-6 in the acute COVID-19 groups; (**B**) IFN-α, (**D**) TNF-α, and (**F**) IL-6 levels in the post-COVID-19 period groups.

In the analysis of plasma cytokine levels, it was observed that patients with the severe form of COVID-19 had higher levels of IFN-α (p = 0.0006; Fig. 2A), TNF-α (*p* = 0.0137; Fig. 2C) and IL-6 (*p* = 0.0071; Fig. 2E) than those with the nonsevere form. Furthermore, individuals with long COVID had higher levels of IFN-α (*p* = 0.0001; Fig. 2B) than those without long COVID symptoms, but there was no significant difference in TNF-α and IL-6 levels between these groups (Fig. 2D and F).

### Correlation of the evaluated inflammatory markers

**Figure 3:**
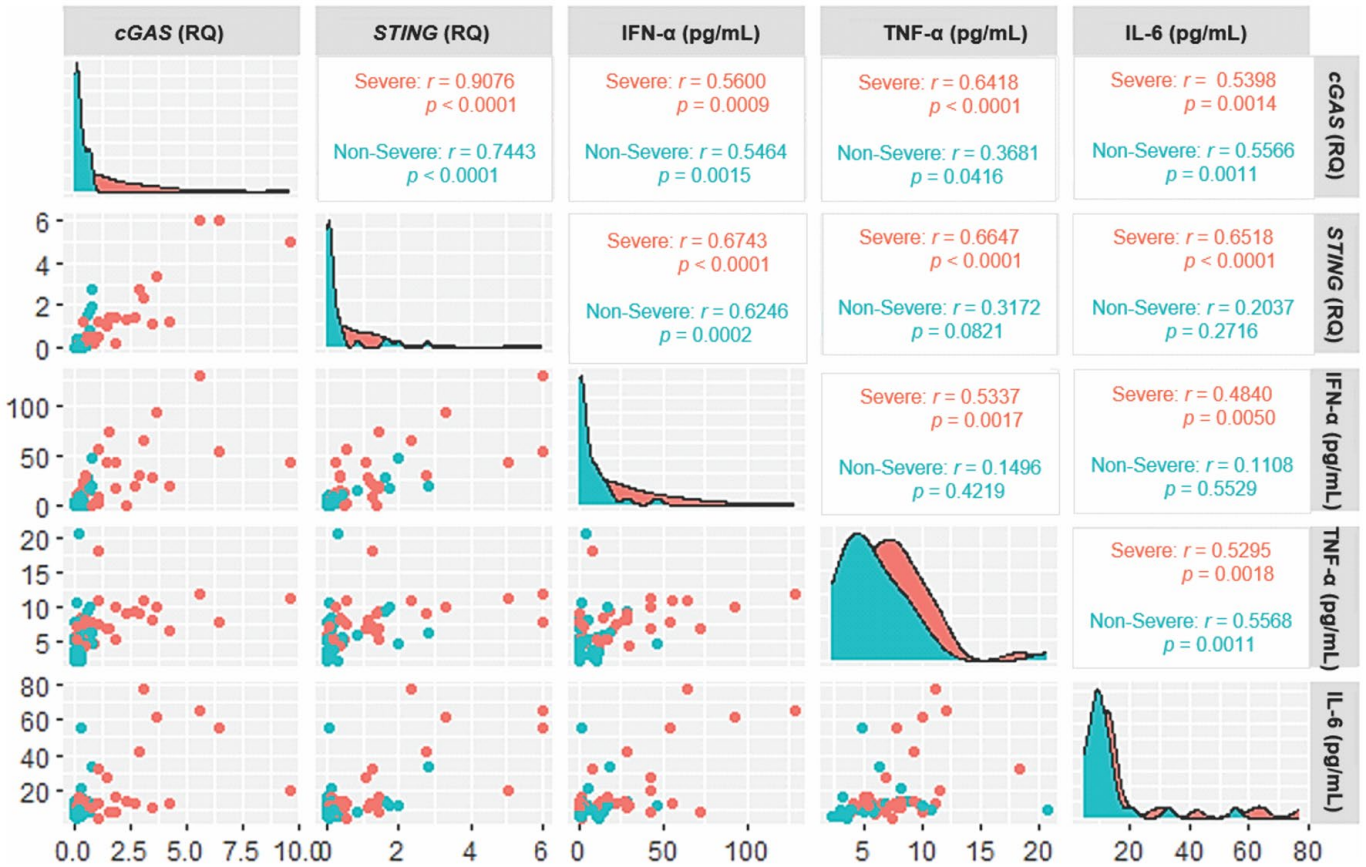
Correlogram of the expression levels of *cGAS* and *STING* and plasma levels of the cytokines IFN-α, TNF-α and IL-6 in patients with acute COVID-19. Scatter plots show the correlation between markers, and density-adjusted curve plots show the distribution of individuals in relation to marker concentration.

In assessing the correlation between the gene expression levels of *cGAS*, *STING* and cytokines in acute COVID-19, a positive correlation was observed between the levels of all markers in the group with the severe form of the disease. A positive correlation with almost all markers was also detected in the group with the nonsevere form, except for *STING* with TNF-α, *STING* with IL-6, IFN-α with TNF-α and IFN-α with IL-6 (Fig. 3).

**Figure 4:**
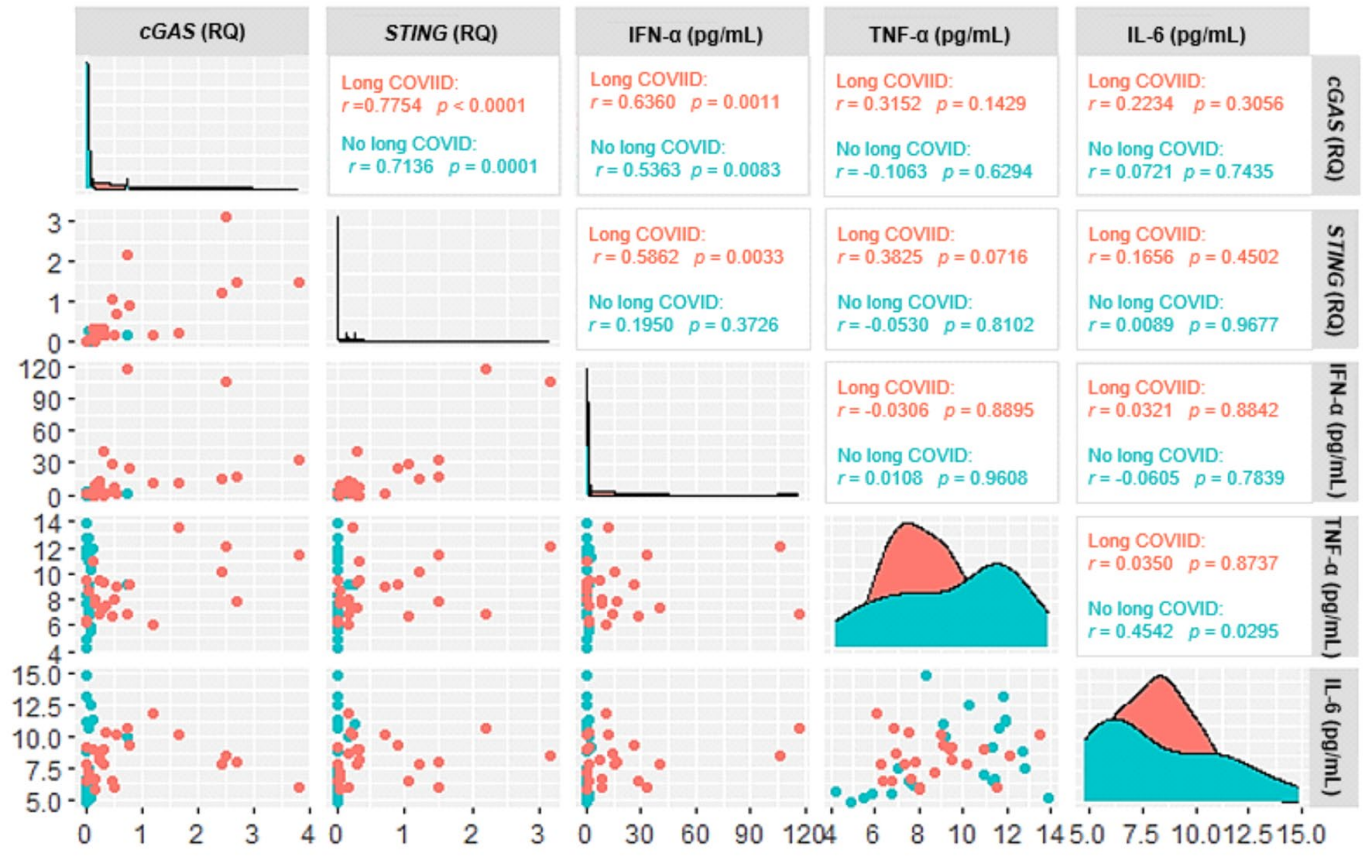
Correlogram of the expression levels of *cGAS* and *STING* and plasma levels of the cytokines IFN-α, TNF-α and IL-6 in individuals in the post-COVID-19 period. Scatter plots show the correlation between markers, and density-adjusted curve plots show the distribution of individuals in relation to marker concentration.

In the group of individuals in the post-COVID-19 period, there was a positive correlation only between *cGAS* and *STING* and *cGAS* and IFN-α in the long COVID and without long COVID groups and between *STING* and IFN-α in the long COVID group (Fig. 4).

**Figure 5:**
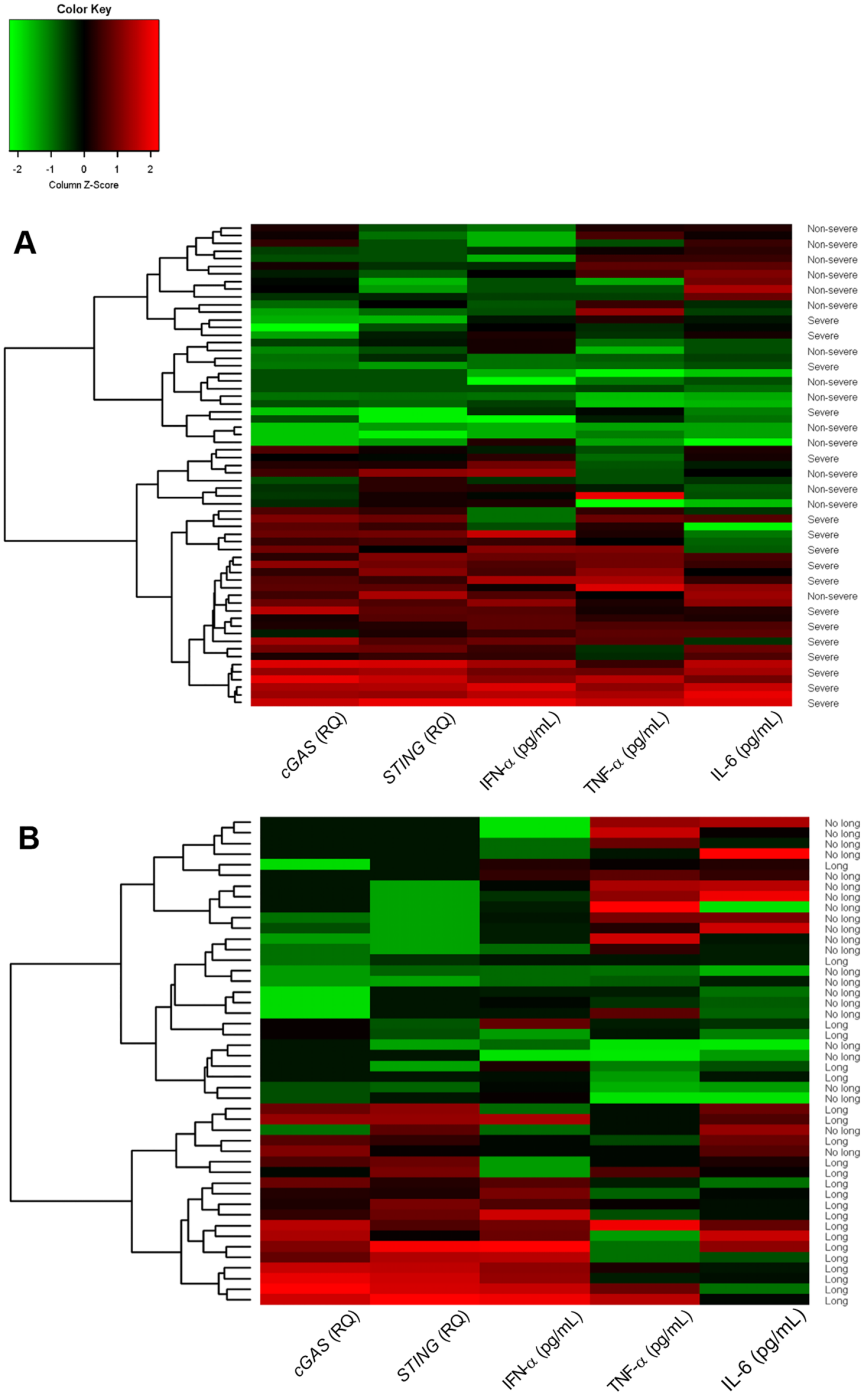
Heatmap of the gene expression levels of *cGAS* and *STING* and plasma levels of IFN-α, TNF-α and IL-6 in the (**A**) acute COVID-19 and (**B**) post-COVID-19 periods.

A heatmap was constructed for joint analysis of the gene expression levels of *cGAS* and *STING* and plasma levels of IFN-α, TNF-α and IL-6 in acute COVID-19 and in the post-COVID-19 period. In acute COVID-19, two groups were observed: one consisting mainly of patients with severe COVID-19, who showed greater levels of gene expression and the cytokines evaluated, and another including patients with nonsevere manifestations of the disease (Fig. 5A). In evaluation of the groups in the post-COVID-19 period, the heatmap illustrated two groups, with the group formed by patients with long COVID having higher gene expression of *cGAS* and *STING* and cytokine IFN-α levels (Fig. 5B).

**Figure 6:**
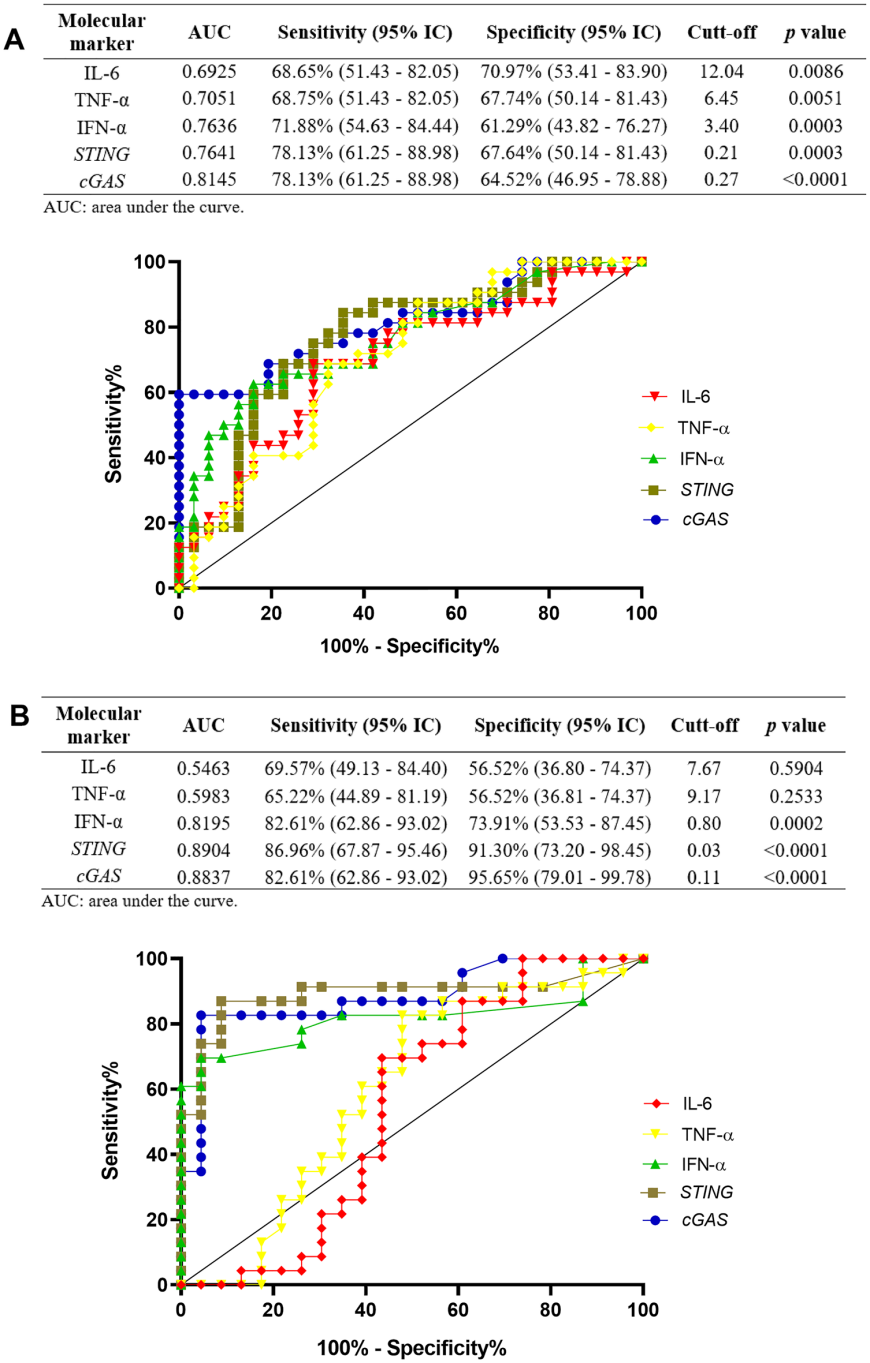
ROC curve parameters of the markers investigated between (**A**) individuals with severe and non-severe manifestations during acute COVID-19 and (**B**) individuals with and without long COVID in the post-COVID period.

The ability of the levels of the investigated markers to differentiate groups in acute COVID-19 and in the post-COVID period was evaluated by the ROC curve. In acute COVID-19, TNF-α, IFN-α, *STING* and *cGAS* showed a performance that varied from fair to good in differentiating severe and non-severe cases, with area under the curve (AUC) ranging from 0.7051 to 0.8145 (*p* < 0.05; Fig. 6A). In the post-COVID period, the IFN-α, *STING* and *cGAS* markers show good ability to differentiate individuals with and without long COVID, with AUC that ranged from 0.8195 to 0.8904 (*p* < 0.005; Fig. 6B).

### Inflammatory markers evaluated in relation to risk factors for COVID-19 and long COVID

The gene expression levels *of cGAS*, *STING* and the plasma levels of the cytokines IFN-α, TNF-α and IL-6 in patients with acute COVID-19 were evaluated according to factors considered risk factors for the severity of the disease, namely, sex, age and comorbidities. However, no association was observed between the levels of any of the markers and male sex, advanced age (> 60 years) or the presence of comorbidities (Table 3).

**Table 3:** Assessment of the gene expression levels of *cGAS* and *STING* and plasma levels of the cytokines IFN-α, TNF-α and IL-6 according to established risk factors for the severity of acute COVID-19.

Variables	*cGAS* (RQ)	*STING* (RQ)	IFN-α (pg/mL)	TNF-α (pg/mL)	IL-6 (pg/mL)
	Median (IIQ)	Median (IIQ)	Median (IIQ)	Median (IIQ)	Median (IIQ)
**Sex**					
Male	0.26 (0.96)	0.33 (1.12)	7.6 (9.3)	6.10 (3.84)	12.96 (7.33)
Female	0.46 (0.86)	0.28 (1.25)	2.6 (14.5)	6.75 (3.88)	11.55 (5.23)
*p*	0.6461	0.7777	0.4507	0.7553	0.481
**Age**					
21–40	0.14 (0.46)	0.11 (0.58)	1.2 (9.3)	7.44 (4.56)	11.39 (5.65)
41–60	0.57 (2.6)	0.56 (1.21)	14.2 (30.8)	6.54 (4.11)	13.01 (17.23)
> 60	0.29 (0.71)	0.17 (0.69)	1.9 (13.8)	5.72 (3.54)	12.93 (7.29)
*p*	0.1964	0.0095	0.0224	0.6396	0.2873
**Comorbidities**					
Present	0.37 (1.22)	0.31 (1.02)	10.7 (24.2)	5.86 (3.27)	13.03 (11.32)
Absent	0.27 (0.72)	0.29(1.20)	3.4 (17.1)	7.05 (4.45)	11.53 (5.28)
*p*	0.2666	0.7268	0.2939	0.0827	0.1658

IIQ: interquartile range. Comorbidities: diabetes mellitus, chronic cardiovascular disease, obesity, chronic kidney disease.

Analysis of the long COVID group showed that there was no statistical difference in the expression levels of *cGAS* and *STING* and the cytokines IFN-α, TNF-α and IL-6 between individuals with different amounts of symptoms and those with the presence or absence of comorbidities (Table 4).

**Table 4:** Assessment of the gene expression levels of *cGAS* and *STING* and plasma levels of the investigated cytokines according to the number of symptoms and the presence and absence of comorbidities in the group with long COVID.

Variables	*cGAS* (RQ)	*STING* (RQ)	IFN-α (pg/mL)	TNF-α (pg/mL)	IL-6 (pg/mL)
	Median (IIQ)	Median (IIQ)	Median (IIQ)	Median (IIQ)	Median (IIQ)
**Number of symptoms**					
2 a 4	0.28 (0.45)	0.05 (0.29)	1.90 (7.14)	8.24 (2.26)	7.85 (0.64)
5 a 7	0.72 (1.50)	0.19 (0.74)	10.50 (17.90)	8.56 (2.88)	8.42 (2.94)
8 a 10	0.35 (0.19)	0.18 (0.80)	21.30 (19.37)	8.24 (2.26)	7.70 (2.49)
*p*	0.5080	0.6097	0.4342	0.9857	0.8519
**Comorbidities**					
Present	0.242 (0.43)	0.139 (0.17)	4.62 (12.12)	8.01 (1.69)	8.06 (3.12)
Absent	0.437 (1.94)	0.297 (0.97)	11.70 (21.60)	8.96 (2.67)	8.01 (1.75)
*p*	0.2382	0.3541	0.5474	0.9899	0.8739

IIQ: interquartile range. Comorbidities: diabetes mellitus, chronic cardiovascular disease, obesity, chronic kidney disease.

## Discussion

Based on increased susceptibility in STING-knockout mice, initial studies on the role of STING in RNA virus infections described the importance of this adapter in controlling different infections^28^. In some infections, proteins produced by RNA viruses promote STING degradation, reducing IFN-I levels and facilitating viral replication^17–20^. STING is important for host defense during RNA virus infection, as its activity can also limit the initial steps of viral mRNA translation^16^.

In the present study, higher levels of *STING* and *cGAS* gene expression and plasma levels of IFN-α, IL-6, and TNF-α were identified in patients with the severe form of acute COVID-19 than in patients with the nonsevere form. A positive correlation between the levels of all evaluated markers was also found for severe COVID-19 patients, a relationship also demonstrated by a heatmap.

In viral infections, IFN-I is important for restricting replication by inducing the expression of IFN-stimulated genes (ISGs) and enhancing the immune response of myeloid cells, NK cells, B cells and T lymphocytes^29^. IFN-I, induced by cGAS-STING, binds to the heterodimeric transmembrane receptor composed of the subunits IFNAR1 and IFNAR2, which activates two proteins, Janus receptor-associated tyrosine kinase (JAK1) and tyrosine kinase e (TYK2), which induce dimerization and nuclear translocation of transcription activator 1 (STAT1) and 2 (STAT2) and binding to IRF9. This complex associates with the IFN-stimulated response elements (ISREs), inducing the production of several ISGs. Some ISGs act in the antiviral response such as MX1, OAS and TRIM and others as positive regulators of IFN signaling, including STAT1 and 2, IRF3, 7 and 9 and cGAS, contributing to the maintenance of INF-I production through the cGAS-STING pathway^30,31^.

TNF-α promotes intercellular communication and regulates cell survival, apoptosis and necroptosis^32^. The synthesis of IL-6 at the injury site leads to a change in homeostasis induced by the production of acute-phase proteins and platelets, which promotes changes in vascular permeability and thrombocytopenia^33^. The actions induced by high levels of IL-6, IFN-α, TNF-α have been associated with persistence and dysfunction of severe COVID-19 and with a greater probability of death^34–36^.

Although STING has been shown to participate in the control of RNA virus infections, including SARS-CoV-2 infection, elevated expression of cGAS and STING in patients with severe acute COVID-19 appears to be a consequence of the intense damage caused to infected cells rather than an attempt to limit virus infection. Domizio et al.^22^ demonstrated that the main cells involved in cGAS, STING and IFN-I dysfunction in COVID-19 are endothelial cells and phagocytes. Infection of endothelial cells by SARS-CoV-2 results in damage and the release of mitochondrial DNA into the cytoplasm, inducing cGAS-STING pathway signaling, IFN-I and inflammatory cytokine production and cell death; in phagocytes, enhanced IFN-I production occurs upon phagocytosis of dying endothelial cells and recognition of the DNA from these cells by cGAS. In cells infected with SARS-CoV-2, cGAS-STING is mainly responsible for the inflammatory cytokine production mediated through NF-κβ activation^21^.

Although the role of cGAS, STING and cytokines in the severity of COVID-19 has been described in other populations, our study includes initial information on the relationship among these components in the population of Brazil, more specifically, in the population of the Brazilian Amazon, a mixed-race population (consisting of whites, blacks and indigenous people)^37^ that presents genetic and cultural differences compared to populations evaluated in other studies. The results of the present study reinforce that, regardless of characteristics specific to the population, cGAS-STING pathway contributes significantly to the severity of COVID-19, as these patients have higher gene expression levels of cGAS and STING as well as the cytokines IFN-α, TNF-α and IL-6 in peripheral blood. This pathway promotes maintenance of a systemic inflammatory state, which induces thromboembolic changes and multiple organ failure, the main causes of death in patients with severe disease^38^.

Patients with long COVID had higher *cGAS*, *STING* and IFN-α levels than individuals who did not manifest any symptoms in the post-COVID-19 period. Although the levels of several inflammatory markers have been associated with the severity of acute COVID-19, the development of long COVID is still poorly understood since the persistence of inflammation is associated with only certain conditions, such as neurological manifestations and insulin resistance^39,40^. In a more general assessment of the disease, patients with long COVID had higher levels of IL-17 and lower levels of the anti-inflammatory cytokines IL-10 and IL-4 (compared to individuals who did not develop the syndrome), which suggests a possible dysregulation of the immune-inflammatory balance^10^.

Since in long COVID there is deregulation of the functions of various systems of the human body^41^ and, possibly, high levels of cGAS-STING can contribute to the development of low-grade inflammatory diseases in various organs such as the heart (cardiomyopathy and myocardial infarction), liver (fat accumulation), kidneys (chronic kidney disease), pancreas (type 1 diabetes mellitus) and brain (ischemic stroke)^43^, the elevated levels of cGAS and STING observed in this study may suggest an important role for these markers in the development and maintenance of long COVID. Although *STING* is essential to promote host defense against viral infections by mediating IFN-I production, chronic activation of STING must be inhibited to prevent the development of inflammatory and autoimmune diseases^42–45^.

Several researchers have struggled to understand the causes of long COVID, which can be disabling. They are developing studies with three main foci, which may explain the possible mechanisms of disease pathogenesis^46^, including changes in blood flow, which can lead to the formation of microclots^47,48^, the persistence of the virus in certain body tissues, mainly in the intestinal mucosa^49^ and the unregulated activation of cells of the immune system^50–52^. Although these mechanisms differ, they converge to a common point, the activation of the inflammatory response.

As the processes in the possible causes of long COVID that are being investigated may be related to the persistence of the virus's genetic material or to cell damage, these components may induce overactivation of innate immune pathways, including activation of cGAS-STING. One of the main causes of inflammation is the activation of STING in phagocytes, mediated by the ingestion of damaged cells, since the DNA of these cells can intensify STING activity and induce greater inflammatory cytokine and antibody production and leukocyte activation against the antigens themselves, leading to the development of autoinflammatory disease. In the case of residual virus, this can directly cause the activation of STING in infected cells or favor the phagocytosis of these cells, which further aggravates the activation of STING in phagocytes^22,53–56^. The presence of pro-inflammatory factors induces the activation of pathways that promote neurodegenerative processes and mitochondrial damage, such as the kynurenine pathway (KP)^57^ which can lead to the activation of cGAS and STING and the maintenance of high levels of IFN-I, contributing to the development of some long COVID symptoms, mainly related to neurodegenerative disorders. KP activation was more prolonged and associated with high levels of IFN-β in individuals who have long COVID with neurological changes^58^.

The results of the present study are relevant to understanding that all possible causes of long COVID that can promote chronic inflammatory activation in certain tissues, which can be mainly mediated by activation of the cGAS-STING pathway and IFN-I production. È possivel que outros mecanismos possam contribuir.

The study provides an initial understanding of the role of the cGAS-STING pathway, as one of the possible mechanisms that may be contributing to the development of morbidities associated with long COVID, nevertheless the presence of a higher frequency of female individuals, of an older age (40 to 60 years old) and with comorbidities in the group with long COVID constituted a limitation of the study, since these characteristics are considered risk factors for the severity of COVID-19 and can influence the development of symptoms in long COVID. However, we emphasize that all individuals in the post-COVID period (with and without long COVID) presented mild manifestations during the COVID-19 phase, suggesting the need for a better assessment of the contribution of these risk factors in the establishment of long COVID.

## Conclusion

The SARS-CoV-2 infection induces immune response activation and cell injury, which can vary in intensity. In acute infection, patients with severe COVID-19 had higher levels of *cGAS*, *STING*, IFN-α, TNF-α and IL-6 expression than patients with nonsevere manifestations of the disease, demonstrating that cGAS and STING activation, which is responsible for inducing IFN-I and proinflammatory cytokine production, contributes to the maintenance of an intense systemic inflammatory state characteristic of severe COVID-19. In long COVID, elevated levels of *cGAS*, *STING* and IFN-α after the resolution of SARS-CoV-2 infection may be a consequence of the development of a possible autoinflammatory disease in some tissues, maintained by cGAS-STING activation.

## Data Availability

The data that support the fndings of this study are available on request from the corresponding author.

## Abbreviations

AMP: Adenosine monophosphate
cGAMP: Cyclic GMP-AMP
cGAS: Cyclic GMP-AMP synthase
COVID-19: Coronavirus disease 2019
GMP: Guanosine monophosphate
IFNAR1: Interferon alpha/beta receptor 1
IFNAR2: Interferon-alpha/beta receptor 2
IFN-α: Interferon alpha
IFN-I: Type I interferon
IL-4: Interleukin 4
IL-6: Interleukin 6
IL-10: Interleukin 10
IRF3: Interferon regulatory factor 3
IRF9: Transcription interferon regulatory factor 9
ISGs: IFN-stimulated genes
ISREs: IFN stimulated response elements
JAK1: Janus receptor-associated tyrosine kinase
MX1: MX dynamin like GTPase 1
OAS: 2'-5'-Oligoadenylate synthetases
PRRs: Pattern recognition receptors
RIG-I: Retinoic acid-inducible gene I
ROC: Receiver operating characteristic curve
SARS: Severe acute respiratory syndrome
SARS-CoV-2: Severe acute respiratory syndrome coronavirus 2
STAT1: Transcription activator 1
STAT2: Transcription activator 2
STING: Stimulator of interferon genes
TBK1: TANK-binding kinase 1
TLR: Toll-like receptor
TNF-α: Tumor necrosis factor alpha
TRIM: Tripartite motif-containing proteins
TYK2: Tyrosine kinase 2
